# Regulatory networks underlying mycorrhizal development delineated by genome-wide expression profiling and functional analysis of the transcription factor repertoire of the plant symbiotic fungus *Laccaria bicolor*

**DOI:** 10.1186/s12864-017-4114-7

**Published:** 2017-09-18

**Authors:** Y. Daguerre, E. Levati, J. Ruytinx, E. Tisserant, E. Morin, A. Kohler, B. Montanini, S. Ottonello, A. Brun, C. Veneault-Fourrey, F. Martin

**Affiliations:** 1 INRA, UMR 1136, INRA-Université de Lorraine, Interactions Arbres/Microorganismes, Laboratoire d’Excellence ARBRE, 54280 Champenoux, France; 2 Université de Lorraine, UMR 1136, INRA-Université de Lorraine, Interactions Arbres/Microorganismes, Laboratoire d’Excellence ARBRE, F-54500 Vandoeuvre-lès-, Nancy, France; 3 Dipartimento di Scienze Chimiche, della Vita e della Sostenibilità Ambientale, Università degli Studi di Parma, Parco Area delle Scienze 23/A, 43124 Parma, Italy; 4 Present address: Umeå Plant Science Centre, Department of Forest Genetics and Plant Physiology, Swedish University of Agricultural Sciences, 901 83, Umea, Sweden; 5 Present address: Hasselt University, Centre for Environmental Sciences, Agoralaan building D, 3590 Diepenbeek, Belgium

**Keywords:** Transcription factors, symbiosis, secreted proteins, transcriptional activator trap assay, yeast, transcriptome, ectomycorrhiza development

## Abstract

**Background:**

Ectomycorrhizal (ECM) fungi develop a mutualistic symbiotic interaction with the roots of their host plants. During this process, they undergo a series of developmental transitions from the running hyphae in the rhizosphere to the coenocytic hyphae forming finger-like structures within the root apoplastic space. These transitions, which involve profound, symbiosis-associated metabolic changes, also entail a substantial transcriptome reprogramming with coordinated waves of differentially expressed genes. To date, little is known about the key transcriptional regulators driving these changes, and the aim of the present study was to delineate and functionally characterize the transcription factor (TF) repertoire of the model ECM fungus *Laccaria bicolor*.

**Results:**

We curated the *L. bicolor* gene models coding for transcription factors and assessed their expression and regulation in Poplar and Douglas fir ectomycorrhizae. We identified 285 TFs, 191 of which share a significant similarity with known transcriptional regulators. Expression profiling of the corresponding transcripts identified TF-encoding fungal genes differentially expressed in the ECM root tips of both host plants. The *L. bicolor* core set of differentially expressed TFs consists of 12 and 22 genes that are, respectively, upregulated and downregulated in symbiotic tissues. These TFs resemble known fungal regulators involved in the control of fungal invasive growth, fungal cell wall integrity, carbon and nitrogen metabolism, invasive stress response and fruiting-body development. However, this core set of mycorrhiza-regulated TFs seems to be characteristic of *L. bicolor* and our data suggest that each mycorrhizal fungus has evolved its own set of ECM development regulators. A subset of the above TFs was functionally validated with the use of a heterologous, transcription activation assay in yeast, which also allowed the identification of previously unknown, transcriptionally active yet secreted polypeptides designated as Secreted Transcriptional Activator Proteins (STAPs).

**Conclusions:**

Transcriptional regulators required for ECM symbiosis development in *L. bicolor* have been uncovered and classified through genome-wide analysis. This study also identifies the STAPs as a new class of potential ECM effectors, highly expressed in mycorrhizae, which may be involved in the control of the symbiotic root transcriptome.

**Electronic supplementary material:**

The online version of this article (10.1186/s12864-017-4114-7) contains supplementary material, which is available to authorized users.

## Background

Ectomycorrhizae (ECM) are symbiotic interactions between plant roots and ectomycorrhizal fungi. The plant provides the fungus with photosynthetic sugars and the fungal symbiont gives back low bio-available mineral elements in forest soils for plants, such as nitrogen and phosphorus [36, 61]. Thus, mycorrhizae are crucial for the growth and health of trees in forest ecosystems. The ability to form mutualistic relationships with ECM fungi is restricted to approximately 20,000 plant species, but the ecological and economical importance of these plants is amplified by their widespread occupancy of terrestrial ecosystems [5, 56, 69]. The ECM fungal colony in forest soils is comprised of three main morphological and functional structures: (i) the extramatrical hyphae, so-called free living mycelia (FLM), prospecting the soil for nutrients and receptive host roots, (ii) the symbiotic ECM root tips and (iii) the fruiting body (FB) [34, 61]. ECM root tips are characterized by the presence of three fungal structural components: (i) a mantle of aggregated hyphae ensheathing the rootlets, (ii) a network of coenocytic hyphae (called the Hartig net) penetrating between epidermal and cortical cells, and (iii) a web of extraradical hyphae which forms an essential connection between the colonized root and soil hyphae prospecting the soil for nutrients and with the hyphae forming the fruiting body [37–42, 49, 61]. Fungal colonization leads to striking morphological changes in the plant host roots. The root system in contact with ECM hyphae displays an increased number of lateral roots, a more pronounced elongation of epidermal cells and an arrest of meristematic growth [6, 15, 16, 70]. ECM ontogenesis is also accompanied by significant alterations of the host plant defense system. In contrast with pathogenic fungi that generally induce strong host defense responses, ECM fungi are able to colonize their hosts while inducing only weak and transient defense reactions. Indeed, plant genes involved in defense responses are upregulated during mantle and Hartig net formation but are repressed at later stages of ECM development as observed in the ECM associations *Paxillus involutus*-*Betula pendula*, *Pisolithus microcarpus*-*Eucalyptus globulus* and *Laccaria bicolor-Populus* [14, 23, 28, 29, 33, 74]. The early stages of ECM development are characterized by the up-regulation of fungal genes involved in cell wall adhesion (e.g., hydrophobins) [52, 57] and remodeling (e.g. polygalacturonases and endo glucanases) [71] as well as signaling such as Mycorrhiza-induced Small Secreted Proteins (MiSSPs) [26, 33, 34, 48]. Among these MiSSPs, the *L. bicolor* 7 kDa MiSSP (MiSSP7) is a symbiotic effector required for controlling the plant jasmonate-signaling pathway, a pre-requisite for fungal colonization [51]. The later stages of ECM development are characterized by the up-regulation of genes involved in carbon and nitrogen metabolism, as well as in mitochondrial respiration [11, 12, 14, 28, 29, 33]. All together, symbiosis development leads to a substantial and coordinated transcriptional reprogramming in both partners [34]. However, the regulatory mechanisms triggering and controlling the expression of fungal and plant signaling genes and the developmental pathways leading to ECM symbiosis are largely unknown.

Transcription factors (TF) are master regulators of gene expression. Positively acting TFs, known as “activators”, generally consist of at least one DNA-binding domain (DBD) and one activation-domain (AD). DBD recognizes and binds sequence-specific DNA elements in the promoter region of target genes whether AD recruits and interacts with the transcriptional machinery. TFs are classified into several families based on conserved folds and structures within their DBDs. ADs, instead, are structurally quite variable and this lack of conservation complicates their bioinformatic identification/prediction [63]. To date, only a single study has addressed the genome-wide profiling of TFs in an ECM fungus [44]. Focusing on the ECM symbiosis between the ascomycete *Tuber melanosporum* and hazelnut (*Corylus avellana*), this study identified multiple mycorrhiza-regulated TFs associated with root cell wall remodeling and fatty acid metabolism. In particular, two orthologs of the *XlnR* activator of genes involved in cellulose and xylan degradation were found to be dramatically upregulated in ECM [44].

The aim of the present work was to identify, classify and functionally characterize from a regulatory point of view potential regulators of symbiosis development within the TF repertoire of *L. bicolor*. Combined genomic and transcriptomic analyses provided a comprehensive view of the *L. bicolor* TF repertoire and its regulation during mycorrhizal development. A transcriptional activator trap (TAT) assay, performed in the yeast *Saccharomyces cerevisiae* [31, 44, 65], was used to functionally validate in silico and gene expression data.

## Results

### Comparative analysis of *L. bicolor* repertoire of predicted TFs

**Fig. 1:**
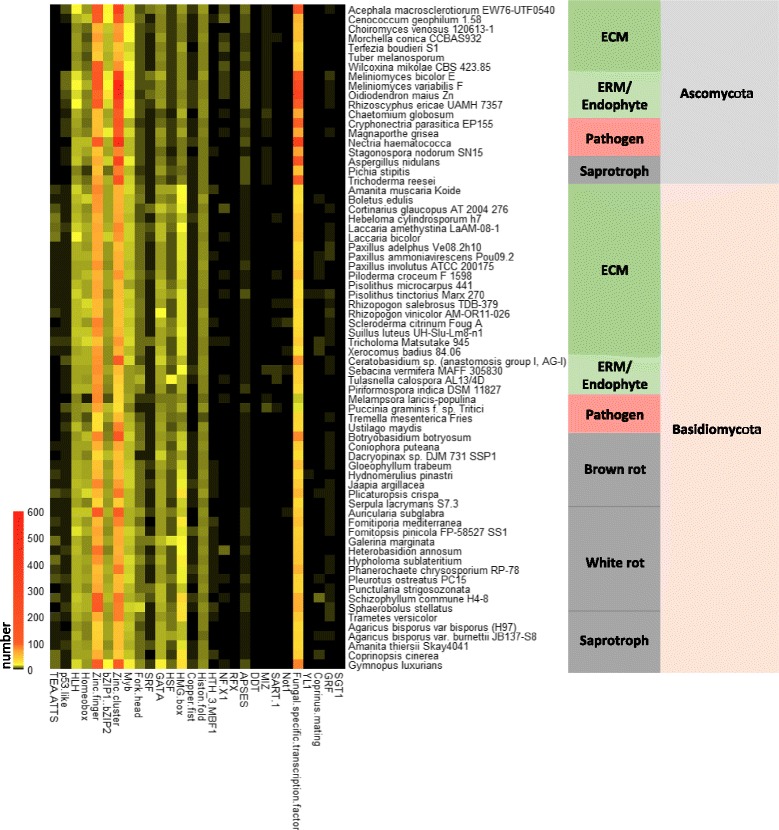
Distribution of TFs in different families in 70 fungal genomes. The heatmap represents the abundance on each TF family in each genome. Abundance levels range from pale to saturated colour (black for absence, yellow for low abundance, red for high abundance). ECM: ectomycorrhiza; ERM: ericoid mycorrhiza; ORM: orchid mycorrhiza

We curated 285 TFs derived from the *L. bicolor* gene catalogue v2.0. As revealed by a DBD-based classification, putative *Laccaria* TFs belong to 28 different classes (Additional file 1: Table S1)*.* This *in silico* generated TF repertoire was compared with the corresponding repertoires of 70 saprotrophic, endophytic, mycorrhizal and pathogenic fungi of diverse clades (Ascomycota and Basidiomycota) [26]. The overall distribution within known TF families is highly similar for the TF repertoires of these fungi and apparently not related to their different lifestyles. Amongst the examined fungi, the three most prevalent TF families are those containing the C2H2 Zinc-finger (PF00096), the Zn2/Cys6 Zinc-cluster (PF00172) and the fungal-specific TF (PF04280) domains (Fig. 1, Additional file 2: Table S2). The number of Zn-cluster and fungal-specific TFs was higher in Ascomycota compared to Basidiomycota. For example, although the predicted proteomes of the ericoid Ascomycete *Meliniomyces variabilis* and the ECM Basidiomycete *Cortinarius glaucopus* are similarly sized (20,389 and 20,377 predicted proteins each), they encode, respectively, 554 and 61 Zn-cluster TFs. In general, Zn-cluster TFs appear to be more represented than Zn-finger TFs in Ascomycota (Fig. 1 and Additional file 2: Table S2), whereas a slight prevalence of the homeobox, GATA, Heat Stress TF (HSF) and High Mobility Group (HMG)-box TF families is observed in Basidiomycota compared to Ascomycota. For instance, eight GATA TFs are encoded by the genome of *M. variabilis*, whereas 13 GATA TF genes are present in the genome of *C. glaucopus* (Fig. 1 and Additional file 2: Table S2).

One hundred eighty three of the 285 predicted *Laccaria* TFs displayed similarities with characterized fungal TFs (Additional file 1: Table S1 lines 5 to 187). Among them, 91 were identified as orthologs of known fungal transcription factors by a BLASTP search conducted against the non-redundant GenBank database, using reciprocal BLAST to infer orthologous relationships (Table 1). These TFs were classified into different functional categories (e.g. cell wall modification, development, cell cycle, metabolism, or response to stress and stimuli) based on the putative or known function(s) of their orthologs (Table 1). Eight additional orthologs of known fungal TFs were retrieved from a BLASTP search using DBD-lacking regulatory proteins as a reference (Additional file 1: Table S1 lines 284 to 291); 94 predicted *L. bicolor* TFs displayed no similarity to any known fungal transcription factor (Additional file 1: Table S1 lines 189 to 282).

**Table 1:** List of putative transcription factors in *Laccaria bicolor* genome

Protein ID gene names and functional classes^a,b^		Putative gene product function^c^
Cell wall		
247,901	LbACE1–1	Repressor of plant cell wall-degrading enzymes
622,364	LbACE1–2	Repressor of plant cell wall-degrading enzymes
293,207	LbRlm1–1	Maintenance of cell wall integrity
302,141	LbRlm1–2	Maintenance of cell wall integrity
Development		
*Sexual development and fruiting body formation*		
**393,192**	**LbSte12α**	**Regulator of fruiting body development**
522,619	LbMcm1	Regulator of pheromone response
668,161	LbNosA	Number of sexual spores, regulator of sexual development
301,103	LbHD1	Mating-type protein
379,291	LbHD2	Mating-type protein
324,166	LbHom1–1	Regulator of fruiting body development; Involved in mushroom tissue formation
399,669	LbHom1–2	Regulator of fruiting body development; Involved in mushroom tissue formation
293,988	LbHom2	Regulator of fruiting body development; Regulation of the formation of the auto-inhibitor and of dikaryon-specific hydrophobins
487,295	LbC2h2	Regulator of fruiting body development; Involved in primordia formation
585,149	LbFst3	Negative regulator of fruiting body development; Inhibits the formation of clusters of mushrooms
585,421	LbNsdD	Regulator of sexual development
644,689	LbExp1	Regulator of the final phase of fruiting-body morphogenesis
308,722	LbFst4	Positive regulator of fruiting body development; Involves in the switch between the vegetative and the reproductive phase and in aggregate formation
685,209	LbGat1	Regulator of fruiting body development; Involved in mushroom tissue formation
300,824	LbItc1	Subunit of ATP-dependent Isw2p-Itc1p chromatin remodeling complex required for repression of a-specific genes, early meiotic genes during mitotic growth, and INO1
**386,478**	**LbPcc1**	**Regulator of sexual development**
381,332	LbPriB	Primordia formation, Regulator of sexual development
705,566	LbCDC5	Regulator of sexual development
313,811	LbMoc3	Regulator of sexual development, ascus formation, and stress response
680,902	LbPrf1	Regulator of pheromone signalling, filamentous growth and pathogenic development
700,295	LbBri1	Regulator of fruiting body development; Regulation of the formation of the auto-inhibitor and of dikaryon-specific hydrophobins
293,563	LbSnf5	Regulator of sexual development
311,495	LbRum1	Repressor for genes regulated by the b mating type locus, involved in spore development
451,323	LbMedA-1	Regulator of sexual and asexual development
483,117	LbMedA-2	Regulator of sexual and asexual development
*Asexual development and basal hyphal growth*		
685,688	LbCol21	Colonial, regulator of hyphal growth
657,026	LbDevR	Required for conidiophore development
**298,274**	**LbAbaA**	**Regulator of conidiation**
292,045	LbCon7	Cell morphology regulator
481,451	LbReb1	Regulator of growth
190,760	LbRsc8	Component of the RSC chromatin remodeling complex essential for viability and mitotic growth
608,593	LbSnt2	Regulator of conidiation, hyphal growth and septation
*Others*		
231,949	LbADA2	All development altered, regulator of basal hyphal growth and asexual and sexual development
Cell cycle		
694,007	LbSwi6	MBF complex, regulator of cell cycle
709,955	LbMbp1	MBF complex, regulator of cell cycle
164,524	LbSep1	Activator for a small subset of mitotic genes involved in septation
476,882	LbFkh2	Regulator of cell cycle
**481,652**	**LbSak1**	**Positive regulator of cAMP-dependent protein kinase-mediated exit from the mitotic cell cycle**
622,520	LbCbf11	Regulator of cell adhesion and cell and nuclear division
691,497	LbSFP1	Regulator of ribosomal protein, biogenesis genes, response to nutrients, stress and DNA-damage, G2/M transitions during mitotic cell cycle and cell size
Metabolism		
*Carbon*		
443,509	LbCreA	Major carbon catabolite repression protein
296,037	LbNrg1	Carbon catabolite repression
399,488	LbAcuk	Positive regulator of gluconeogenesis
567,783	LbAcuM-1	Positive regulator of gluconeogenesis
670,648	LbAcuM-2	Positive regulator of gluconeogenesis
708,062	LbAcuM-3	Positive regulator of gluconeogenesis
708,164	LbRgm1	Positive regulator of monosaccharide catabolism and aldehyde metabolism
308,583	LbCmr1	Regulator of melanin biosynthesis
696,532	LbTrm2	Regulator of methanol-inducible gene expression
*Nitrogen*		
488,576	LbAreA	Major, positively acting, nitrogen regulatory protein
301,157	LbNirA-1	Pathway specific, positively acting nitrate regulatory protein
317,073	LbNirA-2	Pathway specific, positively acting nitrate regulatory protein
**293,242**	**LbGcn4**	**Positive regulator of the transcriptional response to amino acid starvation**
301,697	LbBAS1	Transcription factor, involved in regulating basal and induced expression of genes of the purine and histidine biosynthesis pathways; also involved in regulation of meiotic recombination at specific genes
*Sulfur*		
706,529	LbCBF1	Activator of sulfur metabolism; centromere binding protein
476,130	LbMetR-1	Activator of sulfur metabolism
490,310	LbMetR-2	Activator of sulfur metabolism
*Lipid*		
654,679	LbFarA	Activates transcription of genes required for acetate utilization
573,592	LbOaf3	Negative regulator of fatty acid metabolism
*Others*		
459,853	LbHap2	CCAAT binding complex, subunit B
708,105	LbHap3	CCAAT binding complex, subunit C
694,786	LbHap5	CCAAT binding complex, subunit E
574,778	LbHapX	CCAAT binding complex, subunit X; iron-responsive factor
709,764 + 617,537	LbUrbs1	Negative Regulator of siderophore biosynthesis genes
293,949	LbSfu1	Negative Regulator of Iron Uptake
709,867	LbIec1	Subunit of the Ino80 complex, involved in nucleotide metabolism and phosphate metabolism
*Stress and stimuli response*		
459,072	LbAsg1	Regulator of stress response and drug resistance
699,455	LbHsf1	Heat shock transcription factor
**665,554**	**LbYap1**	**Regulator of oxidative stress tolerance**
582,197 + 625,683	LbSkn7	Response to osmotic and oxidative stress
379,257	LbPacC	Activator of alkaline-induced genes; repressor of acid-induced genes
150,072	LbCrz1–1	Activator of genes involved in stress response
636,734	LbCrz1–2	Activator of genes involved in stress response
681,767	LbMSN4	Activator of genes involved in stress response
607,158	LbZap1	Activator of zinc responsive genes
652,780	LbHxl1	Unfolded protein response
387,518	LbWC1	Light response and circadian rhythm regulator
306,097	LbWC2	Light response and circadian rhythm regulator
636,228	LbMbf1	Transcriptional coactivator involved in DNA replication stress and GCN4-dependent transcriptional activation
442,607	LbXbp1	Stress-induced transcriptional repressor during mitosis, and late in meiosis
Others		
301,089	LbBdp1	Transcription factor, involved in transcription of genes encoding tRNAs, 5S rRNA, U6 snRNA, and other small RNAs
619,068	LbFhl1	Regulator of ribosomal protein (RP) transcription
636,637	LbIIIA	Transcription factor, required for transcription of 5S rRNA
149,540	LbNCB1 subunit alpha	Subunit of a heterodimeric NC2 transcription regulator complex
660,430	LbNCB2 subunit beta	Subunit of a heterodimeric NC2 transcription regulator complex
571,647	LbSql1	General transcriptional co-repressor
667,862	LbAtf2	
309,497	LbDpb4	Subunit of the chromatin remodeling complex ISW2
294,914	LbAbf2	Mitochondrial nucleoid protein
474,585	LbNhp6B	Activator of the RNA polymerase III SNR6 gene
625,238	LbPli1	SUMO E3 ligase involved in centromere and telomere maintenance
669,147	LbSet3	Histone deacetylase involved in the regulation of cytokinesis
702,907	LbSwc4	Subunit of the chromatin-remodeling complexes NuA4 and SWR1
611,756	LbCdc39	Subunit of the CCR4-NOT1 core complex
686,238	LbSnu66	Subunit of the U4/U6.U5 snRNP complex

^a^TFs retrieved from and functionally validated by the TAT screen are in bold. See also Fig. 8

^b^ *L. bicolor* transcription factors were grouped into major functional classes based on homology with functionally characterized TFs from other fungi; gene names were derived from those of the corresponding homologs (see Additional file 1: Table S1 for further sequence information)

^c^Specific putative function of *L. bicolor* TFs as deduced from the known function of their characterized homologs

### TF expression profiling during ectomycorrhizal development

**Fig. 2:**
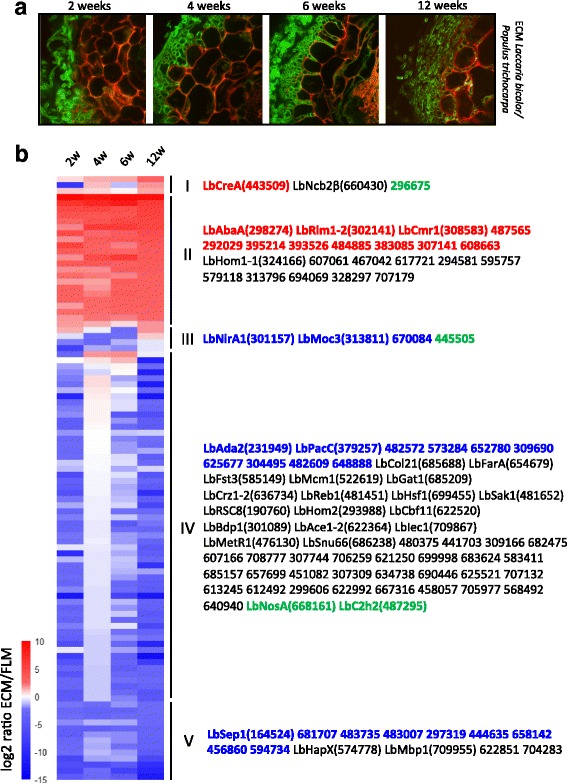
Differential expression of *L. bicolor* TFs during the development of poplar (*P. trichocarpa*) ECM. **a** Laser-scanning confocal microscopy images of transverse sections of *P. trichocarpa* roots 2, 4, 6 and 12 weeks after contact with *L. bicolor* hyphae. Plant root cells are counterstained with propidium iodide and fungal cell walls are revealed using WGA-AlexaFluor 488. Bars indicate 10 μm. **b** Clustering of 100 differentially expressed *L. bicolor* TF transcripts (>2.5-fold; BH, modified *t*-test <0.05) during ECM development (2, 4, 6 and 12 weeks after contact) compared to free-living mycelium (see Additional file 3: Table S3 for the list of transcripts). Log2 transformed data were manually clustered. Each gene is represented by a row of coloured boxes (corresponding to ratio values) and a single column represents each developmental time-point. Regulation levels range from pale to saturated colours (red for induction; blue for repression). White indicates no change in gene expression. Protein IDs are given for each cluster. TFs up- or downregulated during both poplar and Douglas fir ECM development are shown in red and blue, respectively. TFs regulated in an opposite manner in ECM root tips of *P. trichocarpa* or *P. menziesii* are in green

**Fig. 3:**
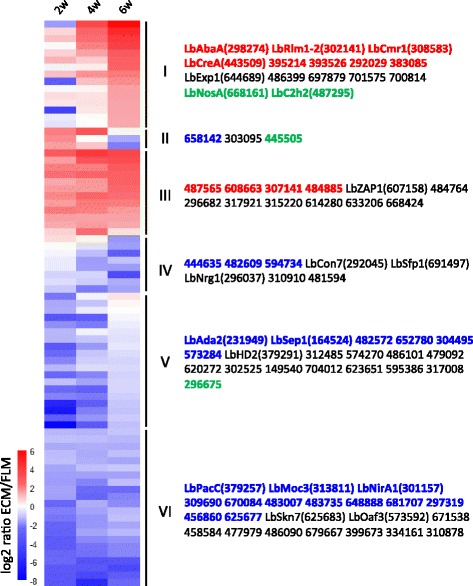
Differential expression of *L. bicolor* TFs during the development of Douglas fir (*P. menziesii*) ECM. Clustering of 79 differentially-expressed *L. bicolor* TF transcripts (>2.5-fold; BH, modified *t*-test <0.05) during ECM development (2, 4 and 6 weeks after contact) compared to free-living mycelium (see Additional file 4: Table S4 for the list of transcripts). Log2 transformed data were manually clustered. Each gene is represented by a row of coloured boxes (corresponding to ratio values) and a single column represents each time-point. Regulation levels range from pale to saturated colours (red for induction; blue for repression). White indicates no change in gene expression. Protein IDs are given for each cluster. TFs up- or downregulated during both poplar and Douglas fir ECM development are shown in red and blue, respectively. TFs regulated in an opposite manner in ECM root tips of *P. trichocarpa* or *P. menziesii* are in green

We profiled the expression of *Laccaria* TFs during symbiosis development in 2-, 4-, 6- and 12-week-old *Populus trichocarpa* and *Pseudotsuga menziesii* ECM root tips [55]. Each of the above time-points corresponds to a distinct ECM developmental stage (Fig. 2a). Two weeks post-contact, *L. bicolor* hyphae colonize the root surface and loosely aggregate onto rhizodermal root cells. After 4 weeks, hyphae ensheath plant roots forming the inner mantle, a multilayer hyphal pseudoparenchyma, in which fungal hyphae penetrate between rhizodermal cells. At 6 weeks, hyphae have formed the Hartig net and reached the cortical cell layer. After 12 weeks, the Hartig net is fully differentiated between cortical cells, whereas rhizodermal cells have collapsed. Douglas fir ECMs are considered to be anatomically differentiated and functional after six weeks, wheras 12 weeks are required to reach the same developmental stage in *P. trichocarpa* ECMs*.* Manual clustering maps of TFs differentially expressed compared to the free-living mycelium (≥ or ≤2.5-fold, corrected *p*-value ≤0.05) in poplar and Douglas fir ECMs are shown in Figs. 2b and 3, respectively.

### Transcription factor expression patterns during *P. trichocarpa* – *L. bicolor* Mycorrhiza development

We manually identified five distinct clusters of differentially TF genes in *P. trichocarpa*- *L. bicolor* mycorrhizae compared to the free-living mycelium stage (Fig. 2, Additional file 3: Table S3). Cluster I corresponds to TFs upregulated during the latest stage of ECM development (6 and/or 12 weeks). They likely regulate the expression of genes involved in ECM functioning and in bi-directional nutrient exchanges. One of them, JGI ID# 443509, is related to the C2H2-type Zinc finger regulator CreA, involved in carbon catabolite repression in *Aspergillus nidulans*. Cluster II comprises TFs upregulated throughout ECM development (at least in 3 over the 4 time-points), suggesting a possible role of these components during the entire course of symbiosis development or even a general role throughout fungal development. Only four of these TFs are homologous to proteins of known function. LbRlm1–2 (JGI ID# 302141) is related to the MADS-box transcription factor RlmA, which regulates cell wall reinforcement in response to physical stress, whereas LbAbaA (JGI ID# 298274) is homologous to AbaA, a TEA/ATTS superfamily TF regulating hyphal growth. LbHom1–1 (JGI ID# 324166) belongs to the HOX homeodomain TF superfamily and LbCmr1 (JGI ID# 308583) is homologous to Cmr1, a regulator of pigment production.

Clusters III, IV and V comprise downregulated TFs. Cluster III contains TF transcripts downregulated at four and six weeks. One of these transcripts codes for LbNirA1 a homolog of the nitrate assimilation pathway activator NirA. Its downregulation might be instrumental to finely tune nitrate metabolism during ECM formation. TFs in cluster IV are downregulated at either early (2 weeks) or late (12 weeks) stages of ECM development. The expression profiles of most of these TFs (46 out of 58) are unique to poplar-*L. bicolor* mycorrhizae. This cluster also contains TFs ressembling known regulators of nutrient (especially lipid) metabolism, such as LbMetR (JGI ID# 476130) and LbFarA (JGI ID# 654679). Several others TFs are related to regulators involved in fruiting body development (LbFst3, JGI ID# 585149; LbC2H2, JGI ID# 487295; LbHom2, JGI ID# 293988), hyphal growth (LbCol21, JGI ID# 685688; LbReb1, JGI ID# 481451)) and stress response (LbCrz1–2, JGI ID# 636734; LbPacC, JGI ID# 379257). TF transcripts downregulated throughout ECM development (at least in 3 over the 4 time-points) are grouped in cluster V, which includes the putative iron responsive factor LbHapX (JGI ID# 574778) and the cell cycle regulator LbMbp1 (JGI ID#709955).

### Transcription factor expression patterns during *P. menziesii* – *L. bicolor* Mycorrhiza development

TF genes differentially expressed during Douglas fir- *L. bicolor* ECM formation were categorized into six distinct clusters according to their expression patterns (Fig. 3, Additional file 4: Table S4). Cluster I comprises TFs upregulated in the latest stage of mycorrhiza development (4 and/or 6 weeks) and similar to the situation previously observed in the case of the poplar symbiosis, it also includes LbCreA (JGI ID# 443509). This suggests that this particular TF may play a key role during the final stages of mycorrhiza development.

Cluster II contains three TFs upregulated during the earlier stages of ECM development (2 and/or 4 weeks), which might be involved in the initial aggregation of the hyphae onto the root surface. One Zn-finger (JGI ID# 445505) protein is unique of *L. bicolor*. Another member of this group (JGI ID# 658142) is a HMG-box transcription factor.

Cluster III is comprised of TFs upregulated throughout ECM development. The activator of the zinc responsive TF LbZap1 belongs to this cluster.

TFs in Cluster IV are downregulated at six weeks. They include LbCon7 (JGI ID# 292045), a C2H2 Zn-finger TF homologous to Con7p, the central regulator of infection-related morphogenesis in the rice blast fungus *Magnaporthe grisea* and LbNrg1 (JGI ID# 296037), the homolog of the carbon catabolite Zn-finger repressor Nrg1 from *Cryptococcus*. Expression of these two genes is unique to Douglas fir- *L. bicolor* mycorrhizae.

TFs downregulated at two and four weeks post-contact are grouped in Cluster V. One of them (JGI ID# 231949) is homologous to Ada2, the regulator of asexual development and basal hyphal growth in *Neurospora crassa*, and another one (JGI ID# 379291) is related to the *Coprinellus disseminatus* mating-type regulator HD2.

Cluster VI contains TFs downregulated throughout ECM development. It includes the homologs of the stress response regulators PacC (JGI ID# 379257) and Skn7 (JGI ID# 625683), the regulator of sexual development, ascus formation and DNA integrity Moc3 (JGI ID# 313811) and the negative regulator of fatty acid metabolism Oaf3 (JGI ID# 573592). Interestingly, the nitrate metabolism regulator LbNirA1, also a member of this cluster, is constitutively downregulated all along Douglas- *L. bicolor* ECM development (Fig. 3, Cluster VI), whereas it was downregulated only at 2, 4 and 6 weeks post-contact, but slightly upregulated in mature mycorrhizae in the case of the poplar-*L. bicolor* interaction (Fig. 2b, Cluster III). It thus seems as if the host plant can regulate the expression of fungal transcription factors involved in nitrate assimilation.

### Commonalities and differences in the regulation of *L. bicolor* TF genes differentially expressed during poplar or Douglas fir ectomycorrhiza development

**Fig. 4:**
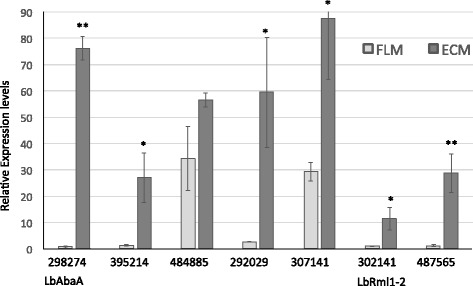
Real-time quantification of TF gene expression in mature *P. trichocarpa* – *L. bicolor* root tips. Gene expression level in ECM is shown for selected TF’s as the fold change compared to free-living mycelium. Mean values (*n* = 3) +/− S.E are represented. Significantly upregulated genes are indicated by * (*p* < 0,05; student T-test) or ** (*p* < 0,01; student T-test)

The expression of 30 and 25 transcription factors is significantly upregulated in Douglas fir and poplar mycorrhizae, respectively. Twelve of them follow the same expression trend in both associations, while 18 and 13 TFs are uniquely upregulated in either Douglas fir or poplar mycorrhizae (Figs. 2b and 3, Additional files 3 and 4: Tables S3 and S4). Transcriptional activators sharing the same expression trend belong to the core set of symbiosis-regulated TFs. They likely regulate the transcription of genes required for symbiosis development. Interestingly, the *Laccaria* orthologs of Cmr1 (JGI ID# 308583), CreA (JGI ID# 443509), AbaA (JGI ID# 298274) and Rlm1–2 (JGI ID# 302141) are part of this group. One gene sharing similarities with the amino acid biosynthesis regulator GCN4 (JGI ID# 395214), and other TFs similar to TFs involved in the regulation of acetate utilization (e.g., the homologs of *Aspergillus nidulans* FacB (JGI ID# 383085) and of *Neurospora crassa* Acu15 (JGI ID# 296682) belong to this group. Seven TFs belonging to this core set of similarly regulated transcription factors were subjected to an independent quantitative RT-Real Time-PCR validation conducted on *L. bicolor – P. trichocarpa* ECM root tips. This confirmed the expression trend revealed by oligo-array analysis for all but one transcription factor (JGI ID# 484885) (Fig. 4) and also highlighted the strikingly high expression levels of the hyphal growth regulator LbAbaA (JGI ID# 298274) [50].

Conversely, expression of 52 and 76 transcription factors is significantly downregulated in Douglas fir- and poplar mycorrhizae, respectively. Twenty-two of these TFs are concordantly regulated in both types of ECM root tips, while 30 and 54 of them are uniquely downregulated in either Douglas fir or poplar mycorrhizae (Figs. 2b and 3, Additional files 3 and 4: Tables S3 and S4). TFs putatively involved in sexual development, fruiting body development and stress response are part of the *L. bicolor* downregulated core regulome, and include orthologs of *Schizosaccharomyces pombe* Moc3 (JGI ID# 313811) and *Aspergillus nidulans* PacC (JGI ID# 379257) as well as homologs to *Ustilago maydis* Prf1 (JGI ID# 482609) and *Schizophyllum commune* Fst4 (JGI ID# 304495). LbNirA (JGI ID# 301157), the ortholog of the *A. nidulans* nitrate assimilation regulator is also downregulated in both mycorrhizae, albeit with a different time-course. Four transcription factors are instead oppositely regulated in Douglas fir and poplar mycorrhizae. Three of these TFs, the putative regulators of sexual development and fruiting body formation LbNosA (JGI ID# 668161), LbC2H2 (JGI ID# 487295) and a TF apparently unique to *Laccaria* (JGI ID# 445505), are selectively upregulated in Douglas fir ECMs, whereas the fourth TF (JGI ID# 296675) is selectively upregulated in poplar ECMs (Figs. 2b and 3).

### TF expression regulation in other mutualistic interactions

**Fig. 5:**
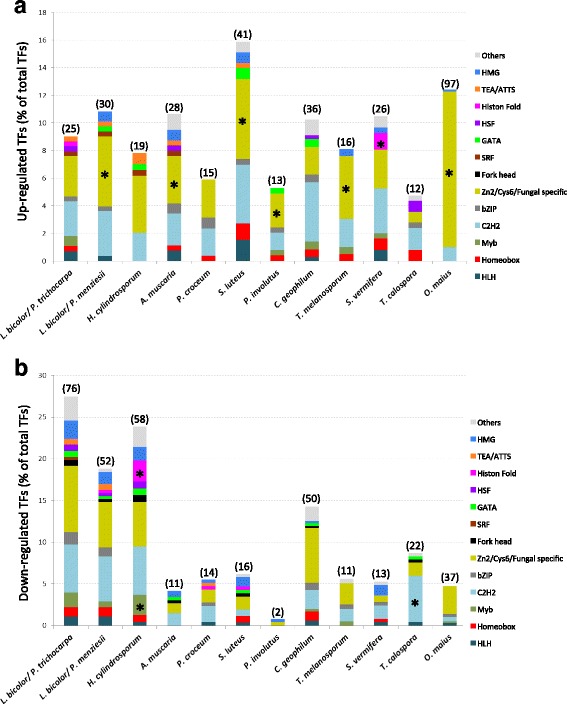
Distribution of differentially-expressed TF gene families in several types of mycorrhizal associations. We retrieved gene expression data of TF-encoding genes from roots colonized by the following species: ECM fungi (*L. bicolor, A. muscaria, H. cylindrosporum, P. croceum, S. luteus, P. involutus, C. geophilum* and *T. melanosporum*), orchid mycorrhizal fungi (*S. vermifera* and *T. calospora*) and one ericoid fungus *O. maius* from Kohler et al. [26]. Differentially-expressed TF genes (≥ 2.5-fold, *p*-value ≤0.05) (**a**, upregualted; **b**, down regulated) in mycorrhizal roots in comparison to free-living mycelium. The histograms show the distribution of TFs from each family as a percentage of the total of TFs in the genome of the corresponding fungi. The total number of regulated TFs for each fungus is indicated between brackets. Stars (*) Indicate families enriched in up-regulated genes compared to the number of these genes in the respective genome (Fisher exact test *p* < 0.05)

*L. bicolor* TF genes regulated during Poplar or Douglas fir ECM development mainly belong to the Zn-cluster-, fungal specific- and C2H2 Zn-finger-superfamilies of DNA binding domains (Fig. 4). To find out whether this is a unique feature of *Laccaria* or a general property of ECM fungi, we searched for differentially expressed TFs (≥2.5-fold, *p*-value <0.05) (Additional file 5: Table S5) in the transcriptomes of the ectomycorrhizal fungi *Amanita muscaria, Cenococcum geophilum, Hebeloma cylindrosporum, Paxillus involutus, Piloderma croceum, Suillus luteus,* and *Tuber melanosporum* [26]. The orchid mycorrhizal fungi *Sebacina vermifera* and *Tulasnella calospora,* and the ericoid mycorrhizal fungus *Oidiodendron maius* were also included in this survey (Additional file 5: Table S5). Depending on the species, upregulated TFs represent 4.8% to 15.9% of the total TF repertoire, and, as in *Laccaria*, they mainly belong to the Zn-cluster-, fungal-specific- and C2H2 Zn -finger-superfamilies (Fig. 5). In half of the examined species, the Zn-cluster- and/or fungal-specific TF families show a statistically significant enrichment in upregulated transcripts compared to their genome abundance (Fisher exact test).

The fraction of downregulated TFs depends on the ECM fungal species and ranges from 0.8% to 27.4% (Fig. 5). No significant enrichment of specific TF families could be identified within downregulated transcription factors, except for the *H. cylindrosporum* symbiotic transcriptome, which is enriched in both histone-fold and Myb TF families, and the C2H2 Zn-finger TF enrichment observed in *T. calospora*.

Assuming that the core set of differentially expressed *L. bicolor* TFs is essential for ECM development, we then examined other ECM fungal genomes/transcriptomes for the presence and mode of regulation of homologous TFs (Additional file 6: Table S6). Interestingly, none of the homologous TF genes upregulated in *L. bicolor* ECM was found to be similarly upregulated in all other symbiotic transcriptomes [26]. However, despite this lack of regulatory overlap, all homologs of upregulated *L. bicolor* TF genes are expressed at above background levels in all ECM fungi and two of them (LbRlm1–2, LbAbaA) are upregulated in all mycorrhizae involving a fungal partner belonging to the Agaricales (i.e., *L. bicolor*, *A. muscaria* and *H. cylindrosporum*). Homologs of LbCreA are also upregulated in *A. muscaria* and *C. geophilum*. Conversely, TFs homologous to the Zn-cluster regulator LbMoc3 (JGI ID# 313811) are concordantly downregulated in the Basidiomycota *A. muscaria*, *H. cylindrosporum*, *S. luteus* and *P. involutus*, and in the Ascomycete *C. geophilum,* suggesting that downregulation of this particular TF is somehow required for ECM development. Altogether, these results suggest that each ECM fungus evolved its own TF network to regulate symbiosis development and functioning. The most notable exceptions are the few TFs (i.e., Rlm1, AbaA and Moc3) that are concordantly regulated in different ECM transcriptomes, which will require more in-depth investigations in order to understand their specific role in mycorrhizal development.

### Functional screening and validation of *L. bicolor* and poplar transcriptional activators in yeast

**Fig. 6:**
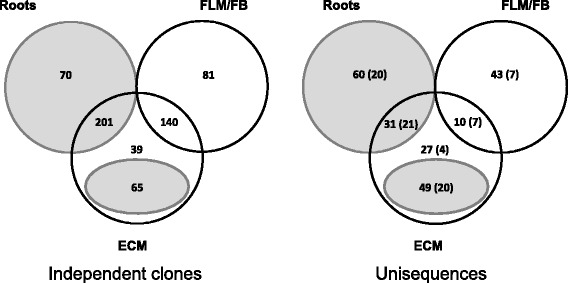
Venn diagram of the number of independent clones (left) and corresponding unisequences (right) isolated from the TAT screening of the FLM + FB, Roots and ECM libraries. The number of DBD-containing clones is shown in brackets. Sequences of plant origin retrieved from the screening of the Roots and ECM library are shown on a gray background

To functionally validate some of the predicted TFs and to uncover potentially new (hard to predict) transcriptional activators, we coupled *in silico* analysis with a heterologous gene transactivation screen, known as transcriptional activator trap (TAT), performed in the yeast *S. cerevisiae* [31, 44, 53, 54, 65]. To this end, three distinct full-length cDNA libraries were prepared from: (i) a mix of free-living mycelium (FLM) and fruiting bodies (FB) (FLM + FB library); (ii) *L. bicolor*/*P. trichocarpa* mycorrhizae of different ages (2, 4, 6 and 12 weeks-old) (ECM library); and (iii) non-mycorrhizal *P. trichocarpa* roots (Root library). Yeast transformants harbouring an in-frame fusion between a *L. bicolor* or *P. trichocarpa* transcriptional activation domain and the DNA-binding domain of the yeast regulator GAL4 were positively selected via reporter gene transactivation assays. Approximately 1.7, 2.0 and 0.8 million colonies were screened for the FLM + FB, ECM and Root libraries, respectively. A total of 596 sequences (196 from the FLM + FB library, 213 from the ECM library, and 187 from the Root library) were found to be capable of activating the expression of three distinct reporter genes and were retained for further analysis. They were organized into 83 contigs and 137 singletons corresponding to a total of 220 unisequences (Fig. 6).

Approximately 63% (140 out of 220) of the retrieved unisequences were of plant origin and the remaining ones were from the fungal partner (Fig. 6). Nearly half of the plant-derived sequences (66 out of 140) encoded either a DNA-binding domain matching the DBDs of *in silico* predicted TFs (61 sequences), or a diverse but still recognizable nucleic acid binding domain (5 sequences). Overall, the TAT assay thus allowed the identification of 61 bona fide plant TFs mainly belonging to the ERF, Myb, NAC, WRKY, Dof and EINL families (Additional file 7: Table S7).

**Fig. 7:**
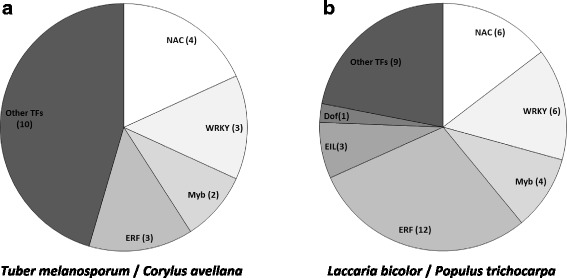
Distribution of plant proteins retrieved from the TAT screening of the ECM library into TF families. The DBD-containing transcription factors of the plant mycorrhizal partner retrieved from the TAT screening of the ECM library are assigned to TF families, either for *T. melanosporum-Corylus avellana* ECM root tips (**a**) and for *L. bicolor-P. trichocarpa* ECM root tips (**b**). Families of TFs related to plant-microbe interactions and pathogen defence [46, 59, 64] are highly represented

As expected, the TAT screen of the ECM library yielded a large fraction of sequences (80 out of 117 unisequences) of plant origin (Fig. 6). Interestingly, half of these putative poplar TFs shared a significant similarity with other plant transcriptional activators known to be involved in plant-microbe interactions, both pathogenic [46, 59, 64] and mutualistic [44] (Fig. 7). On the other hand, only 16 of the in silico identified *L. bicolor* TFs were confirmed and identified by the TAT screen, three of which belong to the core set of symbiosis-regulated TFs. Thirtheen of those genes are within the 20% of the most highly expressed genes at least in one time point of the ectomycorrhiza time-course (80th percentile, data not shown). In addition, the TAT-screen allowed the identification of two DBD-containing activators that were not retrieved from *in silico* analysis (JGI ID# 457991 and JGI ID# 700637) and five activators containing an aspecific nucleic acid binding domain (Table 2). The other 57 TAT-positive putative TFs, all of which lacking a recognizable DBD, are collectively designated as ‘unconventional transcriptional activators’ (see below).

**Table 2:** List of *L. bicolor* TFs, unconventional activators, and putative unconventional activators retrieved from, and functionally validated by, the TAT screen of FLM + FB and ECM libraries

Sequence information				BlastP results			Conserved domains	
seq ID	Protein ID	FLM/FB	ECM	ACC number	Description	Organism	IPR number	Description
*Transcription factors (bearing a recognizable and specific DBD)*								
Contig05	293242	59	10	BAC55240.1	C-Gcn4	*Candida maltosa*	IPR011616	bZIP
ECM-L2_G03	298274	0	1	XP_001388805.1	regulatory protein AbaA	*Aspergillus niger*	IPR000818	TEA/ATTS
ECM-L1_F11	307744	0	1	XP_002173151.1	meiotically upregulated gene product	*Schizosaccharomyces japonicus*	IPR014778	Myb
Contig62	386478	7	0	XP_001830477.1	PCC1	*Coprinopsis cinerea*	IPR000910	HMG1/HMG2
Contig41	393192	3	0	XP_001886200.1	STE12-like	*Laccaria bicolor*	IPR013087	C2H2
ECM-L1_A03	457991	0	1	XP_002910064.1	NWD2	*Coprinopsis cinerea*	IPR000253	Forkhead
Contig40	458057	15	1	XP_001383328.2	ROX1-like HMG-box TF	*Scheffersomyces stipitis*	IPR000910	HMG1/HMG2
Contig65	481652	3	1	XP_003501811.1	RFX2	*Cricetulus griseus*	IPR003150	RFX
FLM-L2_D07	482609	1	0	XP_001828950.2	specific transcriptional repressor	*Coprinopsis cinerea*	IPR000910	HMG1/HMG2
FLM-L2_D05	486090	1	0	XP_003034605.1	expressed protein	*Schizophyllum commune*	IPR001138	Zn2Cys6
Contig46	628355	1	1	XP_567555.1	transcriptional regulatory protein	*Cryptococcus neoformans*	IPR007219	Transcription factor, fungi
FLM-L2_A08	633206	1	0	XP_001368548.1	zinc finger protein 850-like	*Musca domestica*	IPR013087	C2H2
Contig43	640940	3	1	AAC32736.1	Prf1	*Ustilago maydis*	IPR000910	HMG1/HMG2
Contig38	648888	6	3	AAC32736.1	Prf1	*Ustilago maydis*	IPR000910	HMG1/HMG2
ECM-L1_H01	656449	0	1	XP_001819986.2	regulatory protein abaA	*Aspergillus oryzae*	IPR000818	TEA/ATTS
Contig75	665554	2	0	AAS64313.1	Chap1	*Cochliobolus heterostrophus*	IPR011616	bZIP
Contig07	682475	2	0	XP_001399919.1	C6 transcription factor (Mut3)	*Aspergillus niger*	IPR001138	Zn2Cys6
Contig42	700637	16	5	EGO20236.1	hypothetical protein	*Serpula lacrymans*	IPR000433	ZZ Zinc finger
*Proteins containing an aspecific nucleic acid binding domain*								
Contig27	451329	0	2	XP_002911693	CAP-Gly domain-containing protein	*Coprinopsis cinerea*	IPR001878	Zinc finger, CCHC-type
Contig39	585018	2	0	XP_001836279.2	hypothetical protein	*Coprinopsis cinerea*	IPR001606	ARID/BRIGHT DNA-binding domain
Contig45	699941	2	0	XP_001828564.2	hypothetical protein	*Coprinopsis cinerea*	IPR019787	Zinc finger, PHD-finger
FLM-L1_B12	640654	1	0	XP_003507462.1	hypothetical protein	*Coprinopsis cinerea*	IPR018957	Zinc finger, C3HC4 RING-type
ECM-L1_E11	705628	0	1	XP_002172787.1	cps3	*Schizosaccharomyces japonicus*	IPR000571	Zinc finger, CCCH-type
*Unconventional activators with nuclear localization*								
FLM-L3_C01	299583	1	0	XP_001836340.2	TKL/TKL-ccin protein kinase	*Coprinopsis cinerea*	IPR017442	Serine/threonine-PK-like domain
FLM-L2_B06	300643	1	0	CCA73543.1	rec8- related meiotic recombination	*Piriformospora indica*	IPR006910	Rad21/Rec8-like protein, N-terminal
ECM-L2_D08	468224	0	1	XP_001828858.2	Rad21 protein	*Coprinopsis cinerea*	IPR006909	Rad21/Rec8-like protein, C-terminal
Contig70	459401	0	3	XP_001833620.2	ubiquitin-protein ligase	*Coprinopsis cinerea*	IPR000008	C2 calcium-dependent membrane targeting
FLM-L2_F09	610588	1	0	BAG24499.1	rad57	*Coprinopsis cinerea*	IPR013632	DNA recombination and repair protein
Contig72	636246	0	2	CCA72600.1	EDE1-related, endocytosis	*Piriformospora indica*	IPR000449	Ub-associated/transl elongation factor EF1B
ECM-L2_D05	685195	0	1	NP_595780.1	ribosome biogenesis protein Nop6	*Schizosaccharomyces pombe*		
ECM-L1_B01	698517	0	1	XP_002472728.1	60S acidic ribosomal protein P1	*Postia placenta*	IPR001813	Ribosomal protein 60S
FLM-L2_D02	700143	1	0	XP_001828708.2	CMGC/RCK/MAK protein kinase	*Coprinopsis cinerea*	IPR017442	Serine/threonine-PL-like domain
*Putative unconventional activators with intracellular localization*								
Contig03	190404	0	3	XP_001880663.1	aspartic peptidase A1	*Laccaria bicolor*	IPR001461	Peptidase A1
FLM-L1_F07	192523	1	0	XP_001877048.1	tubulin alpha	*Laccaria bicolor*	IPR003008	Tubulin/FtsZ, GTPase domain
FLM-L2_D04	243371	1	0	XP_002912138.1	aconitate hydratase	*Coprinopsis cinerea*	IPR000573	Aconitase A/isopropylmalate dehydratase
Contig36	294384	5	0	XP_568826.1	tubulin binding protein	*Cryptococcus neoformans*		
ECM-L3_B06	324430	0	1	XP_001877591.1	copper transporter	*Laccaria bicolor*	IPR007274	Ctr copper transporter
ECM-L3_A03	327303	0	1	XP_001274183.1	mitochondrial GTPase (YlqF)	*Aspergillus clavatus*	IPR023179	GTP-binding protein
Contig01	444552	4	0	XP_001835217.1	peroxin19	*Coprinopsis cinerea*	IPR006708	Pex19 protein
ECM-L3_D03	521043	0	1	CCA67049.1	related to PDR16- lipid biosynthesis	*Piriformospora indica*	IPR001251	Cellular retinaldehyde-binding
Contig09	583617	8	0	CCA71746.1	related to proteophosphoglycan ppg4	*Piriformospora indica*		
ECM-L2_F10	608638	0	1	XP_001831367.1	gamma-adaptin	*Coprinopsis cinerea*	IPR002553	Clathrin/coatomer adaptor, adaptin-like
FLM-L2_H04	656953	1	0	XP_001837070.1	vacuole protein	*Coprinopsis cinerea*		
FLM-L2_E08	659644	1	0	XP_001839900.1	elongation factor 3	*Coprinopsis cinerea*	IPR015688	Elongation Factor 3
FLM-L2_E09	671307	1	0	XP_001830051.1	peroxisomal targeting signal 1 receptor	*Coprinopsis cinerea*	IPR001440	Tetratricopeptide TPR-1
FLM-L2_D03	695354	1	0	XP_001840019.1	mitochondrial carrier protein	*Coprinopsis cinerea*	IPR018108	Mitochondrial substrate/solute carrier
ECM-L2_E11	703237	0	1	XP_002911229.1	rho GDP-dissociation inhibitor	*Coprinopsis cinerea*	IPR000406	RHO protein GDP dissociation inhibitor
FLM-L2_A07	707485	1	0	XP_002910841.1	guanine nucleotide exchange factor	*Coprinopsis cinerea*	IPR003123	Vacuolar sorting protein 9
ECM-L1_B05	311818	0	1	XP_003037815.1	hypothetical protein	*Schizophyllum commune*		
FLM-L2_F11	321043	1	0	XP_001829416.1	hypothetical protein CC1G_00595	*Coprinopsis cinerea*		
ECM-L2_D01	325350	0	1	P_001837732.1	hypothetical protein CC1G_06938	*Coprinopsis cinerea*		
FLM-L2_H02	390988	1	0	XP_001841219.1	hypothetical protein CC1G_11382	*Coprinopsis cinerea*		
FLM-L1_C04	439929	1	0	EGO18585.1	hypothetical protein	*Serpula lacrymans*		
Contig32	459061	0	3	XP_003026161.1	hypothetical protein	*Schizophyllum commune*		
Contig10	509577	1	1	XP_001882083.1	predicted protein	*Laccaria bicolor*		
FLM-L3_F02	546684	1	0	EGO29111.1	hypothetical protein	*Serpula lacrymans*		
Contig54	549772	4	1	XP_001833470.2	hypothetical protein CC1G_05170	*Coprinopsis cinerea*		
Contig76	576504	5	0	XP_001828840.2	hypothetical protein	*Coprinopsis cinerea*		
Contig37	604174	2	0	XP_001834463.1	hypothetical protein CC1G_02199	*Coprinopsis cinerea*		
Contig24	613652	2	0	XP_001830379.1	hypothetical protein	*Coprinopsis cinerea*		
ECM-L3_B04	622202	0	1	XP_001828856.1	hypothetical protein CC1G_03650	*Coprinopsis cinerea*		
ECM-L2_E05	626440	0	1	NP_587684.1	hypothetical protein	*Coprinopsis cinerea*	IPR019350	RNA polymerase I-specific transcription initiation factor RRN6-like
FLM-L1_G10	634434	1	0	XP_001831375.1	hypothetical protein CC1G_00922	*Coprinopsis cinerea*		
FLM-L2_F03	656382	1	0	XP_001875331.1	predicted protein	*Laccaria bicolor*		
Contig78	680010	5	0	XP_002476516.1	predicted protein	*Postia placenta*		
FLM-L2_E07	686252	1	0	XP_001836432.2	hypothetical protein CC1G_07079	*Coprinopsis cinerea*		
ECM-L3_C02	688275	0	1	XP_001835642.2	hypothetical protein CC1G_03424	*Coprinopsis cinerea*	IPR016021	MIF4-like, type 1/2/3
Contig58	693322	0	4	XP_001830639.2	hypothetical protein CC1G_06905	*Coprinopsis cinerea*	IPR003864	Domain of unknown function DUF221
ECM-L1_B02	693899	0	1	EGO22612.1	hypothetical protein	*Serpula lacrymans*		
FLM-L1_E04	708222	1	0	XP_003036324.1	expressed protein	*Schizophyllum commune*		
Contig68	708574	0	2	XP_001833764.1	hypothetical protein	*Coprinopsis cinerea*		
*Secreted protein*								
ECM-L1_H02	394934	0	1	ZP_01463678.1	sphingolipid ceramide N-deacylase	*Stigmatella aurantiaca*		
Contig44	643792	3	0	XP_001836617.1	hypothetical protein CC1G_06204	*Coprinopsis cinerea*		
FLM-L3_H05	658920	1	0	XP_001830283.1	hypothetical protein CC1G_01919	*Coprinopsis cinerea*		
FLM-L2_G08	660445	1	0	XP_001835021.1	hypothetical protein CC1G_09912	*Coprinopsis cinerea*	IPR018499	Tetraspanin
FLM-L1_G04	688063	1	0	XP_001835466.2	hypothetical protein CC1G_05428	*Coprinopsis cinerea*		
*Secreted proteins with nuclear localization signal (STAPs)*								
FLM-L1_A04	304792	1	0	XP_001840014.1	hypothetical protein CC1G_10398	Coprinopsis cinerea		
FLM-L1_F01	391051	1	0	XP_001840014.1	hypothetical protein	Coprinopsis cinerea		
FLM-L1_D07	455116	1	0	XP_001884865.1	predicted protein	Laccaria bicolor		
Contig64	659547	77	1	AAD01986.1	ras related protein	Laccaria bicolor		

**Fig. 8:**
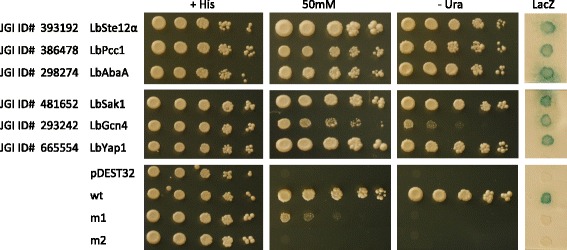
Functional validation of *L. bicolor* transcriptional activators. Representative example of TAT results conducted on the six TFs similar to known function genes. Colonies were isolated from the TAT assay plates and analyzed by serial dilution assays (starting from an OD600 of 1.0) and 2 μl of each dilution were plated on selective plates. Resistance to 50 mM His3 enzyme inhibitor 3-amino-triazole (3-AT) and uracil prototrophy were used to assay the expression of the HIS3 and URA3 reporter genes. For the LacZ (β -Gal) gene reporter assay, 2 μl of yeast cell dilutions (OD600 = 0.1) were spotted on YPD plates overlaid by a nylon membrane, which were then incubated overnight at 30 °C, prior to β -galactosidase assay. Empty pDEST32 vector transformants were used as negative control; wt, m1 and m2 are internal assay controls

Of note, nine of the 16 functionally validated *L. bicolor* TFs resemble known transcription factors regulating development and invasiveness (or pathogenicity). These include two genes closely related to Prf1, the pheromone signalling and filamentous growth regulator of *Ustilago maydis* [21] and two genes similar, respectively, to Pcc1, a regulator of mating and fruiting body formation in *Coprinopsis cinerea* [4, 62], and to Ste12, a TF involved in mating, cell fusion, and in some cases invasive growth regulation in various fungi (reviewed in [7, 73]). Five additional TAT-validated TFs resemble the *Aspergillus nidulans* conidiophore regulator AbaA [1], the *Candida albicans* filamentous growth/virulence regulator Rfg1/Rox1 [8, 25], the oxidative stress tolerance regulator Yap1/Chap1 [10, 30], and the amino acid starvation response transcription factor GCN4 [68] (see Table 2 for further details). The TAT results of a representative subset of these TFs described in Table 1 are shown in Fig. 8.

### Unconventional transcriptional activators

In addition to the validation of a subset of *in silico* predicted TFs, the TAT screen also allowed the identification of 57 putative transcriptional activators lacking a recognizable DBD, and thus designated as ‘unconventional transcriptional activators’ (Table 2). Nine of them resemble known nuclear (e.g., Nop6, Rad21 and Rad 57) or nucleo-cytoplasmic (e.g., Ede 1) proteins, for which a direct or indirect role in transcriptional regulation and/or other nuclear processes (e.g., ribosome biogenesis, double-strand break repair and chromatid cohesion) has been previously documented (Table 2). The remaining TAT-positive sequences code for intracellular proteins without a known transcriptional role, including metabolic enzymes, mitochondrial and peroxisomal proteins, plus 19 conserved hypothetical proteins. Interestingly, nine of these TAT-positive sequences code for predicted secreted proteins, four of which contain a recognizable nuclear localization signal (NLS). We named the latter proteins Secreted Transcriptional Activator Proteins (STAPs) (Table 2). One (JGI ID# 293293) and two (JGI ID# 487613 and JGI ID# 659555) paralogs of the STAPs JGI ID# 391051 and JGI ID# 659547 are present in the *L. bicolor* genome, but only the products of the latter gene models are capable of transcription activation in the yeast system. No conserved shared domain could be recognized in the STAPs, even though some of them contain sequence motifs (an LL motif in JGI ID# 659547 and a coiled-coil region in JGI ID# 391051, JGI ID# 455116 and JGI ID# 659547) known to mediate protein-protein interactions. Of note, the three STAPs (JGI ID# 391051; JGI ID# 304792; JGI ID# 659547) are expressed at fairly high levels in ECM roots (within the 10% of the genes the most highly expressed), suggesting that they may play an important, but as yet unknown role in symbiosis development.

## Discussion

This study provides a genome-wide overview of the repertoire of transcriptional activators of the ECM basidiomycete *L. bicolor* and its regulation during symbiosis development. The latter is a complex multistep process involving a series of sequential morphological changes, with a substantial cell wall remodelling, but also an attenuation (and avoidance) of the host plant defense systems and an extensive metabolic reprogramming.

A total of 285 TFs were *in silico* predicted in the genome assembly v2.0, classified according to their DBDs, and compared with the TFs of 70 fungal species with different lifestyles and taxonomic designations. In accordance with previous data [67], the three most abundant TF families are those containing C2H2 zinc-finger (PF00096), Zn2/Cys6 Zn-cluster (PF00172) and fungal-specific (PF04280) DNA binding domains. The number of Zn-cluster and fungal-specific TFs is higher in species belonging to the phylum Ascomycota compared to species of the phylum Basidiomycota. Prevalence of Homeobox, GATA, HSF and HMG-box TF families is slightly higher in the latter group of fungi. Only one third of the predicted *L. bicolor* TFs is homologous to known TFs. This rather small fraction of homologs may reflect the preponderance of transcription factors functionally characterized in model Ascomycetes such as *N. crassa* and *A. nidulans*.

TF transcript profiling at different stages of Douglas fir and poplar mycorrhizae development identified a core set of differentially expressed transcription factors. One of the most upregulated TFs in this set is LbRlm1–2 (JGI ID# 302141), a MADS box transcription factor involved in cell wall integrity maintenance and invasive growth [17, 24, 72] that is required for pathogenicity in the plant pathogens *Magnaporthe oryzae* [43] and *Botrytis cinerea* [75], as well as in the human pathogen *Aspergillus fumigatus* [58]. In both *M. oryzae* and *A. fumigatus*, *Rlm1* mutants are impaired in invasive growth, with an altered expression of multiple genes coding for cell-wall associated protein. Root apoplastic space invasion by colonizing hyphae is a key step in Hartig net development and in the formation of a symbiotic interface composed of cell wall polysaccharides from the fungus and the host plant [13, 45]. Indeed, multiple transcripts coding for secreted glycosyl hydrolases likely involved in plant and fungal cell wall remodeling are differentially expressed during ECM development in *L. bicolor* [71]. It is thus conceivable to imagine a role for LbRlm1–2 in the regulation of fungal genes coding for glycohydrolytic enzymes shaping the symbiotic interfacial matrix. Rlm1 orthologs are similarly upregulated in the ECMs of the closely related Agaricales *A. muscaria* and *H. cylindrosporum*, whereas the corresponding ortholog in the mycorrizal ascomycete *T. melanosporum* (*TmelRlmA*) is expressed at high levels but not regulated [44]. This suggests that different regulatory strategies may be used by different ECM fungi to accomplish overall similar developmental programs.

Despite the central role of nutrient exchange in ECM symbiosis, expression of TFs known to regulate nutrient uptake and assimilation is generally not regulated during ECM development. Expression of those genes could be very localized or very transiently activated as previously shown for nutrient transporter expression [19]. As we harvested entire ECM root tips, it is thus possible that RNAs corresponding to TF genes are diluted. In addition, it is worth to note that we compare expression level between in vitro free-living mycelium (except for *P. involutus* for which extramatricial patches were harvested) and ECM root tips. However, media for fungal growth and ECM production as well as host-plants are all-different due to different needs from both fungal and plant sides. It could be a reason explaining why we did not find TF-related to nutrition commonly regulated in all symbiotic tissues. Notwithstanding this, the *L. bicolor* homolog of CreA, a transcription factor involved in glucose-mediated carbon catabolite repression in various fungi [9], is strongly up-regulated at a late stage of mycorrhiza formation, when the Hartig net is well differentiated and actively engaged in nutrient exchange and is similarly modulated in *A. muscaria* and *C. geophilum*. In this context, LbCreA, likely represses the expression of genes coding for polysaccharide degrading enzymes such as cellulases and hemicellulases. Upregulation of *LbCreA* at a late stage of ECM development might correlate with the arrest of cell wall remodelling by endoglucanases, polygalacturonate lyases and pectate lyases [71].

Another member of the core set of ECM-regulated TFs is the ortholog of the *A. nidulans* regulatory protein AbaA, which is similarly upregulated in the Agaricales *A. muscaria* and *H. cylindrosporum*, but not in other mycorrhizal fungi. First identified in *A. nidulans* as a regulatory protein required for conidiophore development and maturation [1], AbaA was subsequently shown to be also involved in mycotoxin production, autolysis and cell death [60]. The latter functions suggest a possible role of this TF in the maintenance/turnover of colonizing hyphae as well as in secondary metabolism, at least within the Agaricales.

Three ECM-upregulated TFs display similarities with *N. crassa Acu15* [2] and *A. nidulans FacB* [66], which regulate lipid metabolism in these fungi. Two other related regulators are *M. oryzae* FAR1 and FAR2, which are responsible for differential expression of genes involved in fatty acid β-oxidation, acetyl-CoA translocation, peroxisomal biogenesis, and the glyoxylate cycle in response to lipid availability [3]. Interestingly, the FacB/Acu15 homologues of the mycorrhizal basidiomycetes *A. muscaria, P. involutus, S. luteus* and *P. croceum* are also upregulated in symbiotic tissues, suggesting that controlled expression of fatty acid/lipid metabolism genes is causally linked to ECM development.

Five TF genes downregulated during mycorrhiza formation in *L. bicolor* and in at least four additional ECM basidiomycetes (*A. muscaria, H. cylindrosporum*, *S. luteus* and *P. involutus*) but not in other plant-symbiotic fungi, are homologous to sexual development regulators. The closest matches are *Moc3*, which positively regulates mating efficiency in *S. pombe* [18], *Fst4,* a positive regulator of mushroom development in *Schizophyllum commune* [47]; and *Prf1*, a pheromone response factor coordinating filamentous growth in *U. maydis* [21].

*In silico *TF annotation was at least in part confirmed by the results obtained with the yeast TAT assay. This screen allowed the functional validation of 16 *in silico* predicted TFs as transcriptional activators and the identification of two DBD-containing activators that were not retrieved from *in silico* analysis. The fraction of TAT-validated *Laccaria* TFs is significantly lower (~6%) than that obtained in *T. melanosporum* (20%; [44]) and in the homologous *S. cerevisiae* system (40%, [65]), using similar numbers of assayed transformants. Even though ADs are known to be poorly structured and quite permissive to sequence variations, this relatively low validation rate might be explained by a more marked divergence of basidiomycete (*Laccaria*) TFs from the prototypic amino acid composition of ascomycete (yeast and *Tuber*) activation domains [65]. TAT-validated TFs were generally highly expressed and three of them belonged to the core set of ECM-regulated transcription factors (one upregulated and two downregulated).

In addition to fungal TFs, the TAT screen allowed the functional validation of 61 *in silico* predicted TFs from the host plant *P. trichocarpa*. Most of these TFs belong to the ERF, Myb, NAC, WRKY or EINL families of plant transcription factors, many members of which are known to be involved in the regulation of defense reactions during plant-microbe interaction [46, 59, 64]. Mycorrhiza-induced plant TFs identified by the TAT screen of the ECM cDNA library are likely playing an important role in the control of plant responses to fungal colonisation and thus represent high-priority candidates for future more detailed functional analyses.

The TAT screening also uncovered novel putative fungal activators lacking a DNA binding domain, some of which (e.g., Ede1 and Rsp5) were also identified as “unconventional activators” in *T. melanosporum* [44]. Although quite a few false-positives may be expected due to the presence of a vector-borne NLS that can force the nuclear localization of otherwise cytoplasmic proteins, the occurrence of nuclear moonlighting proteins (capable of a dual function, both in the cytoplasm and in the nucleus) is in line with recent findings in yeast and other micro-organisms [20, 22]. The latter include the ECM ascomycete *T. melanosporum*, in which a cytosolic sulfur metabolic enzyme has been shown to be capable of autonomous nuclear translocation and transcriptional activation in the heterologous host *S. cerevisae* [31].

Of particular interest is the identification, among the “unconventional activators” of the NLS-containing Secreted Transcriptional Activator Proteins. These are reminiscent of the *L. bicolor* effectors Mycorrhiza-induced Small Secreted Proteins [33, 53, 54], one of which (MiSSP7) has been shown to be secreted by the fungus, imported into plant cells through endocytosis and relocalized to the nucleus, where it interacts with, and negatively regulates, the jasmonate pathway co-receptor JAZ6 to attenuate host defence responses [51]. Similar to MiSSPs, the expression levels of the STAPs genes are particularly elevated in ECM root tips. In contrast with MiSSPs, however, STAPs are expressed at high levels also in free-living mycelium, suggesting a role for these proteins also in vegetative growth. Another characteristic MiSSPS and STAPs have in common is their lack of orthologs in other mycorrhizal or non-mycorrhizal fungi. The sole exception was *Laccaria amethystina*, a close relative of *L. bicolor*, indicating that STAPs are largely unique, and at best clade*-*specific, proteins. Although additional data are required to better delineate the in vivo function of these proteins, it is tempting to speculate that STAPs may represent a novel class of intercellularly trafficked transcriptional regulators that may act on the symbiotic plant partner, but, perhaps, also on surrounding fungal hyphae and on other rhizosphere microbes.

## Conclusions

We identify *L. bicolor* TF regulome, which contains TF-genes commonly regulated in both ectomycorrhizal root tips with two distinct host p﻿lants, one Angiosperm (*Populus*) and one Gymnosperm (Douglas fir). We provide evidence that each ECM fungi use its own set of TFs to integrate exogenous signals and drive transcriptional changes leading to ECM development. This could be explained that despite similar morphological changes to occur, the signals could be highly variable in their nature requiring specific TFs to combine them. Nevertheless, keeping in mind the extreme species-specificity of the regulators employed by different ECM fungi to implement an ultimately quite similar symbiotic program, ECM-regulated TFs identified in this work require further investigation, particularly with regard to the gene networks they control. The TAT screening also allowed the isolation of protein without a DNA binding site. These components, especially those without a prior record of nuclear localization and activity, represent a significant outcome of this work. This points to the existence of an as yet largely unexplored set of “hard to predict” transcriptional regulators, as the new class of NLS-bearing, secreted proteins, so-called STAPs. STAPs were not up regulated during ECM development and their constitutive high expression may reflect a role in controlling competing rhizospheric microbes. Therefore, STAPs could be a new class of effectors secreted by *L. bicolor* to control the host plant gene expression during ECM development and/or competing rhizospheric microbes.

## Methods

### Microorganism and plant material

*Saccharomyces cerevisiae* strain MaV103 (MATa, *leu*2–3112, *trp*1–901, *his*3∆200, *ade*2–101, *gal*4∆, *gal*80∆, *SPAL*10*::URA*3, *GAL*1::*lac*Z, *HIS*3UASGAL1*::HIS*3@*LYS*2, *can*1R, *cyh*2R) was propagated on YAPD medium (1% yeast extract, 2% peptone, 2% glucose, and 40 mg/L adenine) and cultured at 30 °C.

The *Populus trichocarpa – Laccaria bicolor* and *Douglas* fir- *Laccaria bicolor* ECM root tips were obtained as described in [55].

### Bioinformatic predictions and annotations of TF

Fungal genome assemblies and annotations used in this study are available via the JGI fungal genome portal MycoCosm (http://jgi.doe.gov/fungi) (Additional file 2: Table S2). Each DNA-binding domains (DBD) of eukaryotic transcription factors is related to a pfam domain, used in combination with other DBD-characteristiscs to classify transcription factors (TFs). Searching for proteins having a TF specific pfam domain retrieved TFs from *L. bicolor* and the other fungi. Functional annotations were performed using BLASTP (https://blast.ncbi.nlm.nih.gov/Blast.cgi) search against the nr database from the National Center for Biotechnology Information (NCBI) with a threshold e-value of 10^−5^. Best hits associated with experimentally characterization and/or publications were reported. The presence of NLS and coiled coil regions were inferred with PSORT II prediction at http://psort1.hgc.jp/. N-terminal signal peptides and cleavage sites were predicted using SignalP at http://www.cbs.dtu.dk/services/SignalP/. To further confirm that the sequence similarity with experimentally characterised TFs was not limited to the highly conserved DBD, BLAST analysis was also performed masking the query DBD (see [32, 44] for further details). Results summarising the outcome of this analysis (i.e. the query is still similar to experimentally characterised TFs) are shown in Additional file 1: Table S1.

### Transcript profiling of TF-encoding genes in ECM

To retrieve expression data on ectomycorrhizal root tips for different mutualistic interactions, we used the complete expression datasets published and available as series at the Gene Expression Omnibus at the National Center for Biotechnology Information website (http://www.ncbi.nlm.nih.gov/geo/). *L. bicolor- P. trichocarpa, L-bicolor- Douglas* fir time course experiments are available under GSE62225 and GSE62226 accession numbers [55]. Expression data for *A. muscaria- Populus tremula x tremuloides*, *H. cylindrosporum- Pinus pinaster*, *P. involutus- Betula pendula*, *P. croceum- Quercus robur*, *S. luteus- Pinus sylvestris* ectomycorrhizal root tips, *T. calospora- Serapias vomeracea* mycorrhizal protocorms, *O. maius- Vaccinium myrtillus* mycorrhizal roots and *S. vermifera- Arabidopsis thaliana* mycorrhizal roots are published in [26] and available at GSE63947 accession number. Expression data of *C. geophilum- P. sylvestris* are available under GSE83909 accession number [48]. Expression data of *T. melanospsorum*- hazelnut ECM are available under GSE17529 accession number [35]. In all cases, only transcription factors significantly regulated (up- or down-regulation ≥ or ≤2.5 fold, Benjamini Hochberg, modified t-test *p*-value <0.05,) were considered for the comparative analysis. When microarray data were used, only transcription factors with an expression ≥200 (arbitrary units) in at least one of the condition tested were retained. For *L. bicolor* time course, clustering of the transcription factors with significant regulation was performed manually, according to their expression pattern throughout the time-course and explained for each cluster in the results section. Data were normalized using log transformation with a Log base of 2 and the heat maps were generated using the pheatmap package in R [27].

### Quantitative real-time qPCR

cDNA was generated from 500 ng total RNA samples using the i-Script cDNA reverse transcription kit from Biorad. Real-time PCR reactions were prepared using SYBR Green Kit (Biorad) including 10 ng cDNA and 300 nM forward and reverse primer in each reaction. PCR was performed in the RotorGene (Qiagen) with the standard cycle conditions: 95 °C for 3 min; 40 cycles at 95 °C for 15 s and 65 °C for 30 s, followed by a melting curve analysis (temperature range from 65 °C to 95 °C with 0.5 °C increase every 10s) A no template control, containing H_2_O instead of cDNA, was included. Transcript abundance was normalized using *L. bicolor* histone H4 (JGI ID# 319764) and ubiquitin (JGI ID #446085) -encoding genes. Stability of the reference genes was validated using GeNorm. The ratios of expression between two conditions were calculated using Pfaffl et al. 2011 method. The primer pairs for each gene are in Additional file 8: Table S8. The amplification efficiency (E) was experimentally measured for each primer pair (Additional file 8: Table S8). Three independent biological replicates were run in duplicate for each experimental condition.

### cDNA libraries construction

cDNA libraries were constructed as reported in Plett et al., [53, 54]. Briefly, total RNA (500 μg per sample) was prepared from *L. bicolor* fruiting bodies (FB), free-living mycelium (FLM) of *L. bicolor* (strain S238 N) grown in P5 liquid Pachlewski medium for 3 weeks, *L. bicolor* mycorrhiza root tips and non mycorrhiza roots tips of *Populus trichocarpa* during a time course of symbiosis development (2, 4, 6 and 12 weeks). RNA extraction was performed using the RNeasy Plant Mini Kit (Qiagen) (for mycorrhiza and non-mycorrhiza root tips, buffer RLC containing 20 mg ml^−1^ of PEG 8000 was used), followed by a DNase I treatment. Total mRNA were purified using Oligotex columns (Qiagen) and used to build FB + FLM and ECM + Roots cDNA libraries with the CloneMiner cDNA Library Construction Kit (Invitrogen), starting from 2 μg and 500 ng of purified total RNA, respectivley. Entry cDNA libraries were then transferred to the yeast-expressible pDEST32 vector using the Gateway system (ProQuest Two-Hybrid System kit, Invitrogen).

### Transcriptional activator trap assays

Yeast strain MaV103 harboring three Gal4-dependent reporter genes (*LacZ, HIS3 *and *URA3*), was transformed with 20 μg of each pDEST32 cDNA library and plated onto twenty Petri dishes (150 mm diameter) on selective medium (SD-Leu-His) containing 25 mM 3-amino-1, 2, 4-triazole (3AT). About 1.7, 2, and 0.8 million colonies were screened for *Laccaria* FLM/FB, ECM, and Root library, respectively. Colonies growing on SD-Leu-His +3AT were individually transferred to 384-well SD-Leu plates using a 384-multipinner device (V&P). To eliminate false positive and evaluate the strength of the interaction, clones collected for their growth on –His + 3AT in the initial screen were replicated to test for the expression of 3 reporter genes (*LacZ*, *HIS3* and *URA3*) as described [44, 53, 54]. Transcriptional activator trap-positive clones are defined as colonies that scored positive to the three reporter genes (about 200 colonies for each library). Corresponding DNA sequences were first trimmed and used as queries for a BLASTX search against the *L. bicolor* and *P. trichocarpa* proteomes at the NCBI and a gene model was assigned to each sequence if the identity was >95%. We checked manually DNA sequences corresponding to the same gene model and included them in contig sequences.

## Additional files

Additional file 1: Table S1. List of *L. bicolor* TFs identified *in silico* displaying similarities with characterized TFs (183 proteins); sharing similarities with predicted or hypothetical proteins with no proved role as transcriptional regulators (94 proteins); sharing similarities with proteins identified as transcriptional regulators without DBD (8 proteins). General information of protein is given (e.g., protein ID from both *L. bicolor* genome version 1 and version 2, length of the polypeptide, TF name, EST support). Accession number, Description, Species, % identity, E-value and Score are related to BLAST results using *L. bicolor* protein as query against fungal NCBI database. m.c. = manually curated. Best Blast reciprocal hit = “YES” indicates that the TF was positive to the Best reciprocal hit analysis. BLAST masked DBD = “YES” indicates that *L. bicolor* TF was still similar to the characterized protein after masking its DBD in a BLAST search. See Methods section for a detailed description. TFs retrieved from and functionally validated by the TAT screen are in bold. (XLSX 48 kb)

Additional file 2: Table S2. Number of proteins within each TF family for 70 fungal genomes. Data used to generate Fig. 1. (XLSX 32 kb)

Additional file 3: Table S3. Gene expression of *L. bicolor* TF-encoding genes that are significantly regulated during ECM development with *Populus trichocarpa* at 2, 4, 6 and/or 12 weeks. Data used to generate Fig. 2b. (XLSX 26 kb)

Additional file 4: Table S4. Gene expression of *L. bicolor* TF-encoding genes that are significantly regulated during ECM development with *Pseudotsuga menziesii* at 2, 4 and/or 6 weeks. Data used to generate Fig. 3. (XLSX 18 kb)

Additional file 5: Table S5. List and expression level of TF-encoding genes significantly up or downregulated in various mutualistic interaction (expression ratio > 2.5 folds and FDR or BH modified t-test < 0.05): **A**. *A. muscaria- Populus tremula x tremuloides.* **B** *. C. geophilum- Pinus sylvestris* **C.** *H. cylindrosporum- Pinus pinaster* **D.** *P. involutus- Betula pendula* **E.** *P. croceum-Quercus robur* **F.** *S. luteus- Pinus* **G.** *T. melanospsorum*- *C. avellana* **H.** *S. vermifera- Arabidopsis thaliana* **I.** *T. calospora- Serapias vomeracea* **J.** *O. maius- Vaccinium myrtillus*. (XLSX 66 kb)

Additional file 6: Table S6. List of *L. bicolor* “core” TF orthologues genes in ECM fungi (*A. muscaria; H. cylindrosporum; P. involutus, P. croceum, S. luteus, C. geophilum* and *T. melanospsorum*). The shaded rows indicate orthologues (using Reciprocal Blast Hit) of *L. bicolor* TFs, which are up or downregulated in symbiotic tissues. (XLSX 25 kb)

Additional file 7: Table S7. List of *P. trichocarpa* TFs, unconventional activators and putative unconventional activators retrieved from, and functionally validated by, the TAT screen of ECM and Roots libraries. For each gene, additional information (e.g. number of clones retrieved, name and other characteristics of the most informative best hit obtained from a BLASTX search carried out in GenBank, IPR domain if any) is reported. (XLSX 27 kb)

Additional file 8: Table S8. List and sequences of primers used for qPCR. (XLSX 9 kb)

## Abbreviations

3AT: 3-amino-1,2,4-triazole
DBD: DNA-binding domain
ECM: Ectomycorrhiza
TAT: Transcriptional activator trap
TF: Transcription factor
